# Exploring the Effects of Residence Time on the Utility of Stable Isotopes and S/C Ratios as Proxies for Ocean Connectivity

**DOI:** 10.1021/acsearthspacechem.3c00018

**Published:** 2023-07-08

**Authors:** Eva E. Stüeken, Sebastian Viehmann, Simon V. Hohl

**Affiliations:** † University of St Andrews, School of Earth & Environmental Sciences, Bute Building, Queen’s Terrace, St Andrews, Fife KY16 9TS, United Kingdom; ‡ Department of Lithospheric Research, University of Vienna, Josef Holaubek-Platz 2, 1090 Vienna, Austria; § Institute of Mineralogy, Leibniz University Hannover, Callinstraβe 3, 30167 Hannover, Germany; ∥ State Key Laboratory of Marine Geology, Tongji University, Siping Road 1239, Shanghai, 200092, P.R. China

**Keywords:** nitrogen isotopes, sulfur isotopes, nonmarine environments, residence time, Miocene, stromatolites, Paratethys

## Abstract

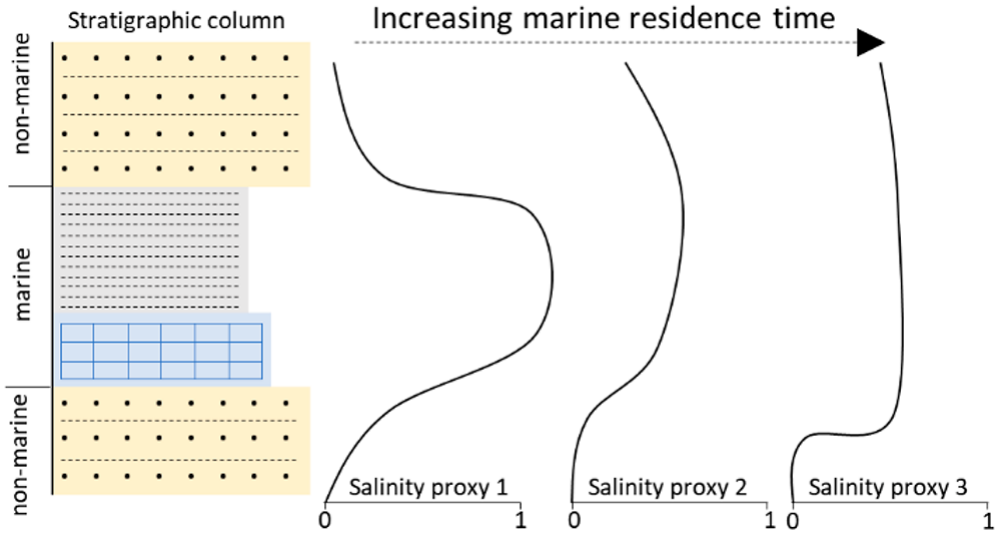

Various geochemical proxies have been developed to determine if ancient sedimentary strata were deposited in marine or nonmarine environments. A critical parameter for proxy reliability is the residence time of aqueous species in seawater, which is rarely considered for proxies relying on stable isotopes and elemental abundance ratios. Differences in residence time may affect our ability to track geologically short-lived alternations between marine and nonmarine conditions. To test this effect for sulfur and nitrogen isotopes and sulfur/carbon ratios, we investigated a stratigraphic section in the Miocene Oberpullendorf Basin in Austria. Here, previous work revealed typical seawater-like rare earth element and yttrium (REY) systematics transitioning to nonmarine-like systematics. This shift was interpreted as a brief transition from an open marine depositional setting to a restricted embayment with a reduced level of exchange with the open ocean and possibly freshwater influence. Our isotopic results show no discernible response in carbonate-associated sulfate sulfur isotopes and carbon/sulfur abundance ratios during the interval of marine restriction inferred from the REY data, but nitrogen isotopes show a decrease by several permil. This observation is consistent with the much longer residence time of sulfate in seawater compared with REY and nitrate. Hence, this case study illustrates that the residence time is a key factor for the utility of seawater proxies. In some cases, it may make geochemical parameters more sensitive to marine water influx than paleontological observations, as in the Oberpullendorf Basin. Particular care is warranted in deep time, when marine residence times likely differ markedly from the modern.

## 1 Introduction

Over the past few decades, several geochemical proxies have been developed to distinguish between marine and nonmarine environmental conditions during the deposition of ancient sedimentary strata. Examples of these include organic carbon-to-sulfur ratios,^1^ strontium/barium ratios, and boron/gallium ratios in shales,^2^ as well as rare earth element (REE) patterns, ^143^Nd/^144^Nd isotope systematics, and ^87^Sr/^86^Sr ratios in chemical sediments such as carbonates or cherts.^3−7^ Applications of these proxies have provided important constraints on the interpretation of several sedimentary units in the rock record, especially in the Precambrian, where fossils that are diagnostic of either freshwater or seawater conditions are absent.^3−5,8^ One caveat in the application of geochemical proxies is the difference in residence time of various proxy elements in seawater (globally or within the regional environment), which impacts their ability to track geologically short-lived transitions between marine and nonmarine conditions in the stratigraphic record. Such rapid transitions may, for example, be associated with sea level changes in response to glacial–interglacial cycles. Proxies whose residence time in the water column exceeds the time scales of such relatively rapid fluctuations are likely to show a different response compared to elements with much shorter residence times. Residence time considerations may therefore impact the interpretation of geochemical data and should guide sampling strategies.

The concept of residence time is well established in isotope geochemistry;^6,7,9^ however, it has not been well explored for salinity proxies that rely on elemental abundance ratios or stable isotopes to investigate connectivity of a given basin to the global ocean. This study is designed to fill this gap for traditional stable isotopes and their elemental abundances (carbon, nitrogen, sulfur). We focus on a case study of the Middle Miocene Oberpullendorf Basin in Austria, a sub-basin of the Paratethys Sea, where previous work identified a stratigraphically short (ca. 60 cm) interval of restricted marine conditions with potential freshwater input in an otherwise open marine basin.^10^ This restricted interval was preserved in stromatolitic carbonates and most conspicuously reflected by shale-normalized (subscript SN) REY_SN_ systematics that lacked the characteristic features of seawater, including pronounced positive La_SN_, Gd_SN_, and Y_SN_ anomalies and heavy REY_SN_-to-light REY_SN_ enrichment (i.e., elevated Yb_SN_/Pr_SN_ ratios). Bracketing strata did show these characteristics. Here, we capitalized on this existing and geochemically well-characterized sample set from Viehmann et al.^10^ to test the response of carbon-to-sulfur ratios and the stable isotopic ratios of carbonate-associated sulfate, total reduced sulfur, organic carbon, and total reduced nitrogen to the proposed fluctuations in ocean connectivity that have been inferred from REY data.

The average sulfur isotope ratio (δ^34^S = [(^34^S/^32^S)_sample_/(^34^S/^32^S)_VCDT_ – 1] × 1,000, where VCDT = Vienna Canyon Diablo Troilite) of modern seawater sulfate (+21.0 ± 0.06 ‰^11^) is markedly higher than that of average riverine sulfate (+4.4 ± 4.5‰^12^), and sulfate concentrations differ by over 2 orders of magnitude (28 mM versus 0.16 mM^12^), meaning that distinct isotopic signatures may be expected in underlying sediments during prevailing marine or nonmarine conditions. Sulfate has a long marine residence time of nearly 9 million years in modern oceans, which is significantly longer than the global ocean mixing time of ca. 1500 years,^13^ and hence, sulfate concentrations and isotope compositions are homogeneous across the world’s oceans today. In contrast, particle-reactive elements like REY and strongly bioreactive species like nitrate have residence times that are lower than the global ocean mixing time^14^ and are therefore impacted by local conditions and short-term variations.

In the case of nitrate, the concentrations of modern rivers are difficult to interpret because they have been impacted by pollution and fertilizers;^15^ however, despite this artificial enrichment, the concentrations are lower (5 μM^15^) than in contemporaneous seawater (31 μM^16^). Hence, less nitrate should be available during nonmarine intervals. The global distribution of marine N isotopic compositions of seawater and sediments (δ^15^N = [(^15^N/^14^N)_sample_/(^15^N/^14^N)_air_ – 1] × 1,000) shows a strong mode at 5–6 ‰^17,18^ with a total range from 2 to 16 ‰.^19^ Sediments archive the isotopic composition of the overlying water column.^17^ Again, modern rivers are impacted by pollution; thus, their measured average isotopic composition of +7 ‰^15^ may not represent natural conditions. In contrast, average Holocene lake sediments record an average value of +2.2 ‰ (range 0–7 ‰^20^), which would be unaffected by anthropogenic pollution. This relatively low value compared with marine sediments likely reflects a relatively higher abundance of diazotrophs (N_2_-fixing organisms) in nitrate-depleted freshwater. Although there is significant local variability in both marine and nonmarine nitrogen isotope values, the average Holocene nonmarine composition is distinct from marine sediments. Hence, distinct isotopic signatures can be expected from open marine versus nonmarine or brackish sediments. The marine residence time of nitrate is 3,000 years, i.e., close or only slightly longer to that of REY.^14^ Therefore, we have a theoretical basis for using both sulfur and nitrogen isotopes to explore how proxies with widely differing residence times respond to short-lived restricted-marine conditions with potential freshwater influence, such as they have been inferred for the Oberpullendorf Basin based on REY data.^10^ In addition, we also measured organic carbon isotopes (δ^13^C = [(^13^C/^12^C)_sample_/(^13^C/^12^C)_VPDB_ – 1] x 1,000, where VPDB = Vienna Peedee belemnite) which may record ecological shifts during fluctuations in ocean connectivity.

## 2 Geological Setting

**Figure 1:**
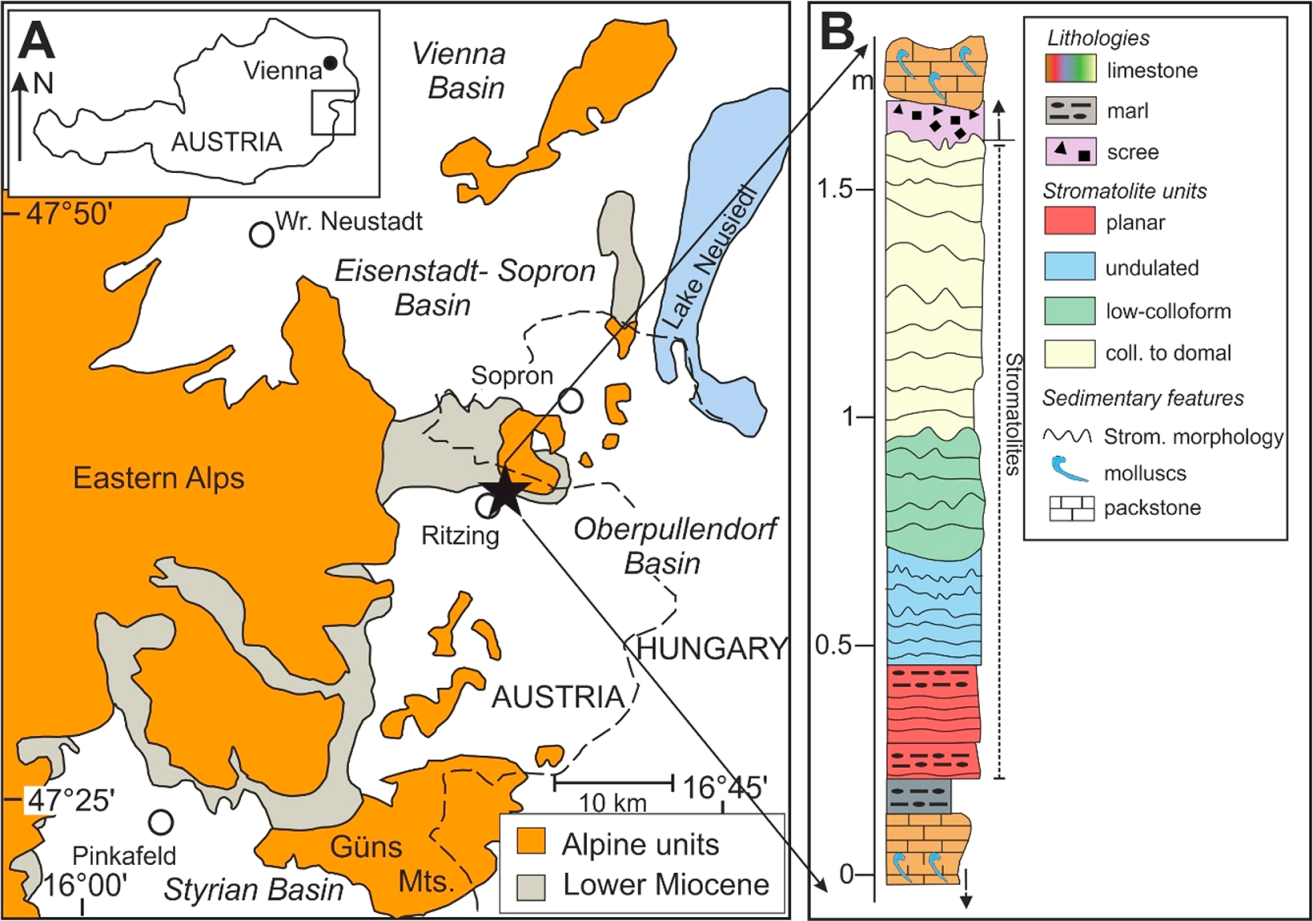
(A) Simplified geological map of the Oberpullendorf Basin. The black star shows the location of the Rabenkopf section near the village Ritzing encountering Middle Miocene stromatolitic limestones of the Badenian salinity crisis. (B) Stratigraphy of the stromatolite-bearing units of the Rabenkopf section, representing paleoenvironments at the top of the second sedimentary cycle during the middle Badenian. Modified from Harzhauser et al.^21^ and Viehmann et al.^10^

Middle Miocene stromatolitic limestones of the Oberpullendorf Basin crop out at the Rabenkopf section (N47°37′0.155″, E16°30′08.53″) near the village Ritzing close to the Austrian–Hungarian border (Figure 1A). The Rabenkopf section was described sedimentologically, petrographically, and geochemically in detail by Harzhauser et al.^21^ and Viehmann et al.^10^ In short, chemo-clastic sediments of this section were deposited at the northern margin of the Oberpullendorf Basin, a western sub-basin of the Parathethys Sea. Marine deposition in this area started during the Badenian (Langhian and early Serravallian), reflected by three consecutive marine depositional cycles, and ended during the late Serravallian.^21,22^ The first cycle, which is only poorly exposed at the study site and was not sampled, can be identified as a Langhian in age due to characteristic foraminifera^23^ and is composed of fossil-rich marls, sands, and limestones that reflect a marine transgression.^21^

Sediments of the second cycle, which was the focus of this study, were deposited during the middle Badenian. This cycle mostly consists of fossil-rich sands and corallinacean-bearing limestones, thought to represent shallow marine conditions. It also includes a roughly 1.4 m-thick package of stromatolitic carbonate that is depleted in macro-fossils. The stromatolites at the base of the Rabenkopf section represent sediments deposited at the end of the second cycle. They show a planar morphology and evolve to undulated, low-colloform and domal appearances near the stratigraphic top (Figure 1B).^10,21^ Our samples are taken from this 1.4 m-thick stromatolitic package.

This interval is thought to correlate with the onset and persistence of the Badenian salinity crisis, which lasted for 200–600 kyr and is expressed elsewhere in Paratethian Sea in the form of evaporite deposits.^24,25^ In the Oberpullendorf Basin, evaporites are absent or have at least not yet been observed, possibly indicating that the water column remained undersaturated with respect to halite and gypsum. Foraminifera of the species *Ammonia beccarii* have been described from the stromatolitic carbonates by Harzhauser et al.,^21^ who interpreted them as evidence of increased salinity due to basin restriction. Today, this species occurs in hypersaline lagoons as well as in brackish waters.^26,27^ In fact, *Ammonia beccarii* is famous for its ability to thrive under a range of environmental conditions and endure rapid fluctuations in salinity and temperature.^26,27^ The occurrence of *Ammonia beccarii* could thus be consistent with the trace element data from Viehmann et al.,^10^ which revealed a deviation from marine conditions over a few decimeters in stratigraphic thickness within the planar stromatolite beds shortly above the base of the section. The REY data may even indicate a freshwater influence during this interval, meaning that conditions were potentially brackish, which could match the appearance of *Ammonia beccarii*.

However, *Ammonia beccarii* persist throughout the stromatolite interval, whereas the REY data suggest a return toward more open-marine conditions in the upper half of the section.^10^ The REY data thus show greater variability and suggest strong fluctuations in water chemistry that are not reflected in the fossil record. This discrepancy between geochemical and paleontological proxies remains to be resolved. The absolute time span for the restricted-marine interval inferred from REY data is difficult to constrain, because the section appears to include depositional hiatuses in the form of temporal subaerial exposure of the stromatolites,^21^ meaning that the total duration of the Badanian salinity crisis (200–600 kyr) is likely only incompletely preserved. In any case, conditions throughout the stromatolitic interval were evidently hostile to macroscopic life, possibly indicating a highly restricted setting in the Central Paratethys.^28^ It is also possible that the relatively rapid transitions between nonmarine and marine conditions indicated by trace element data^10^ were exerting stress on macrofauna, such that microbial life became dominant and *Ammonia beccarii* was able to thrive, given its tolerance to a wide range of conditions.^26,27^ In this case, the geochemical and paleontological data would be reconcilable.

The carbonates of the second depositional cycle are followed by siliciclastic sediments (clays and sands) of the third depositional cycle, which were not sampled in this study. These siliciclastic sediments were deposited during the late Badenian (early Serravallian) and suggest a reflooding of the southern area of the Oberpullendorf Basin.^21^

We obtained off-cuts from the same stromatolite hand specimens and sampling regions as described in Viehmann et al.^10^ Each stromatolite sample comprised multiple laminae, which was necessary to have enough material for analyses. The samples were prepared for analyses of carbonate-associated sulfate, total reduced sulfide, organic carbon isotopes, bulk nitrogen isotopes, and carbonate-hosted trace elements (see Supporting Information for details).

## 3 Results

### 3.1 Isotopic Trends

**Figure 2:**
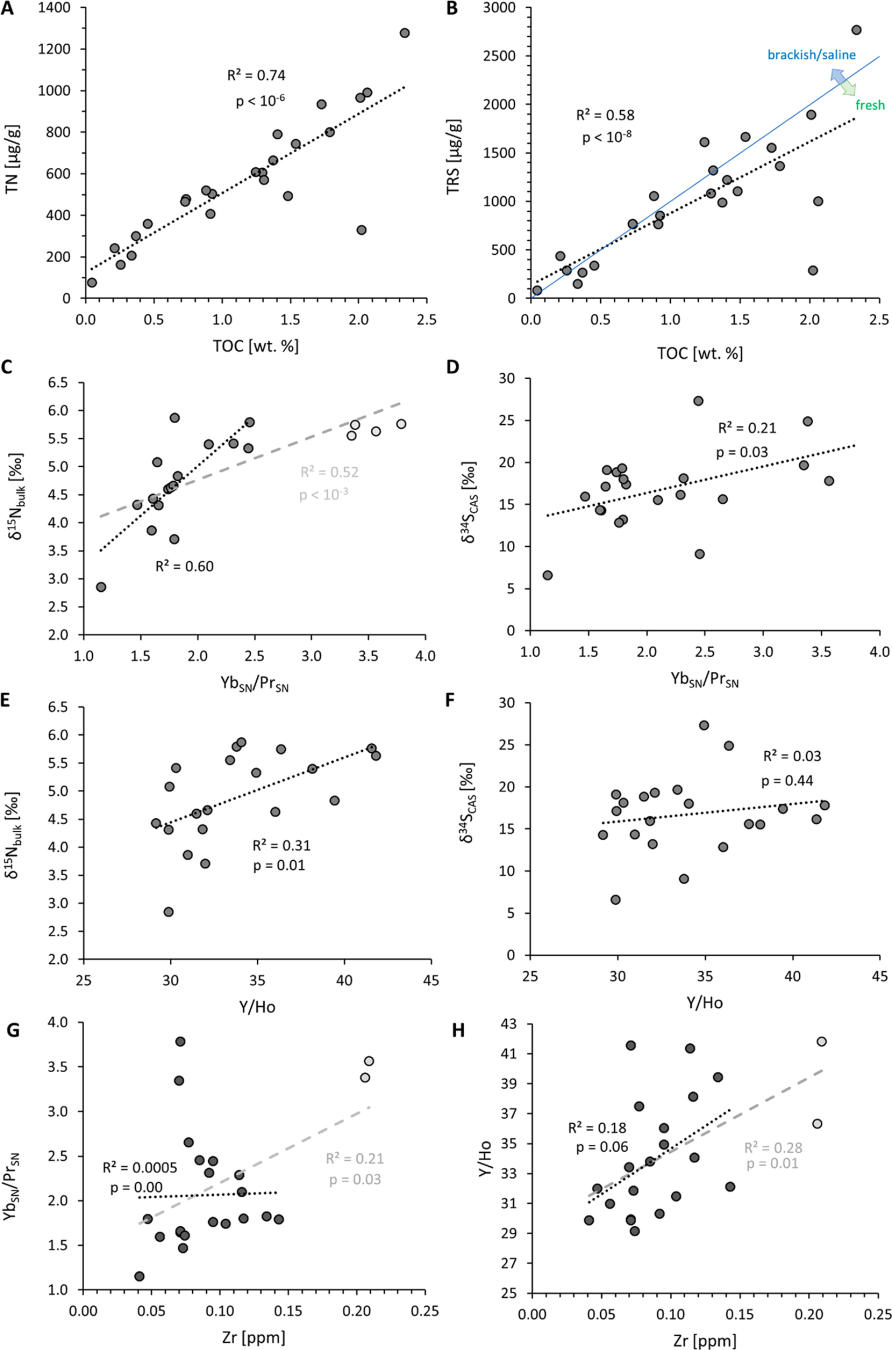
(A) Total nitrogen versus total organic carbon; (B) total reduced sulfur versus total organic carbon; (C) δ^15^N_bulk_ versus shale-normalized Yb/Pr ratios of carbonates; (D) δ^34^S_CAS_ versus shale-normalized Yb/Pr ratios; (E) δ^15^N_bulk_ versus Y/Ho ratios of carbonates; (F) δ^34^S_CAS_ versus Y/Ho ratios of carbonates. (G) Shale-normalized Yb/Pr ratios versus zirconium abundance in carbonates; (H) Y/Ho ratios versus zirconium abundances in carbonates. Total abundances in panels (A) and (B) refer to the decarbonated residues. The threshold between marine/brackish and freshwater conditions in panel B is taken from Wei and Algeo.^2^ It corresponds to a TRS/TOC ratio of 0.1 by mass. Metal abundances in panels C–H are for carbonate leachates. The gray trend lines in panels C, G, and H apply to all data points in the figure; the black trend line applies onto those data points shown in black. The gray data points are statistical outliers by comparison to the remaining data set.

**Figure 3:**
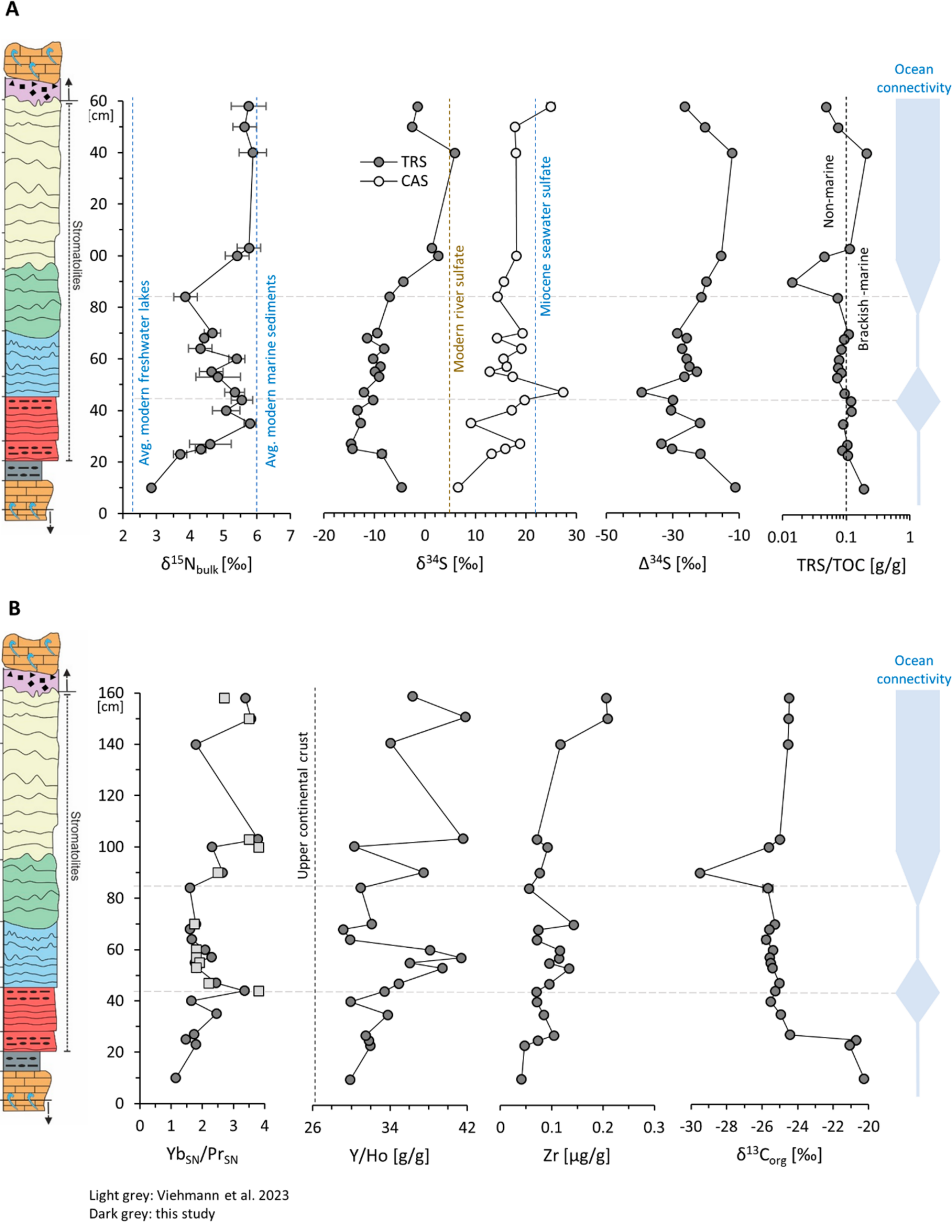
Stratigraphy of (A) Nitrogen and sulfur isotope data (Δ^34^S = δ^34^S_TRS_ – δ^34^SCAS) and TRS/TOC ratios and (B) trace element and carbon isotope data. Lithostratigraphy on the left margin is the same as that in Figure 1. In Panel B, shale-normalized Yb/Pr ratios from Viehmann et al.^10^ are compared to the new acetic acid data from this study, showing good agreement in overall trends. In Panel A, The Miocene seawater sulfate value is taken from Paytan et al.,^29^ modern river sulfate is from Burke et al.,^12^ modern freshwater sedimentary δ^15^N is from McLauchlan et al.,^20^ and modern marine sedimentary δ^15^N is from Tesdal et al.^17^ Dashed horizontal lines in both panels are drawn at those levels that correspond to the maximum Yb_SN_/Pr_SN_ value at 44 cm and the minimum Yb_SN_/Pr_SN_ value at 84 cm, using the data from this study. Ocean connectivity as inferred from the data is discussed in the text.

All studied samples have a high carbonate content with a minimum of 93 wt % and moderate amounts of total organic carbon (TOC), total nitrogen (TN), and total reduced sulfur (TRS) in the decarbonated residues (Table 1). One outlier with comparatively low TOC, TN, and TRS abundances is the nonstromatolitic, bottom-most sample of the section. TOC and TN are strongly correlated (r^2^ = 0.74, *p* < 10^–6^, Figure 2A) with a small TN-axis intercept of 126 μg·g^–1^, indicating that the N in these samples was largely derived from organic matter with a consistent C/N ratio (average 22 ± 5 mol·mol^–1^, with one outlier of 7 at the base and one outlier of 72 at 90 cm). TRS and TOC are also moderately correlated (r^2^ = 0.58, *p* < 10^–8^, Figure 2B), suggesting that, as for N, reduced sulfur was buried in strong association with biomass. TRS/TOC ratios scatter around a mean of 0.10 ± 0.04 g·g^–1^ with no stratigraphic trend (Figure 3).

**Table 1:** Carbon, Nitrogen, and Sulfur Isotope Data Generated in This Study^a^

Position [cm]	Carb. [wt %]	TOC [wt %]	SD	Δ^13^C_Org_ [‰]	SD	TN [mg/kg]	SD	Δ^15^N_Bulk_ [‰]	SD	TRS [mg/kg]	SD	Δ^34^S_Trs_ [‰]	SD	Δ^34^S_Cas_ [‰]	SDio	Trs/Toc [G/G]
158	94.8	2.06	0.07	–24.47	0.00	990	17	5.75	0.52	1001	34	–1.46	0.04	24.88	0.72	0.05
150	98.5	0.45		–24.50		360	0	5.63		337		–2.53		17.80	0.26	0.07
140	98.4	0.21	0.00	–24.54	0.09	241	5	5.87	0.41	438	1	5.92	0.26	18.01		0.21
103	99.5	0.26	0.03	–25.00		162	9	5.76		289		1.39				0.11
100	99.3	0.33	0.07	–25.62		206	13	5.41		152	16	2.66	0.11	18.11		0.05
90	97.1	2.02	0.04	–29.50	0.01	329	12			288		–4.29		15.61		0.01
84	99.1	0.37	0.03	–25.68	0.29	301	11	3.86		269		–7.01		14.34		0.07
70	96.1	1.54	0.00	–25.28	0.03	744	15	4.66	0.24	1667		–9.41		19.31		0.11
68	99.3	0.93	0.02	–25.58	0.01	504	9	4.43	0.05	852	1	–11.47	0.08	14.30		0.09
64	98.7	0.91	0.02	–25.77	0.20	406	2	4.31		765	5	–8.09	0.26	19.11		0.08
60	99.4	1.79	0.08	–25.38	0.12	800	23	5.40	0.24	1366		–10.30		15.55	0.06	0.08
57	99.3	1.48	0.06	–25.56		492	25			1105	51	–8.80	0.22	16.14	0.64	0.07
55	99.0	1.29	0.01	–25.53	0.13	606	5	4.63	0.35	1083	6	–9.89		12.83	0.07	0.08
53	97.9	1.37	0.00	–25.40	0.09	664	3	4.83	0.67	987		–9.08		17.39	0.01	0.07
47	93.1	2.01	0.02	–25.01	0.03	965	10	5.33	0.29	1895		–12.11		27.34		0.10
44	99.5	2.34	0.02	–25.25	0.08	1278	25	5.55	0.33	2770		–10.23		19.69		0.13
40	98.5	0.88	0.02	–25.52	0.08	519	8	5.08	0.42	1056	21	–13.38	0.61	17.14	0.01	0.09
35	99.7	1.73	0.13	–24.95	0.08	934	52	5.79	0.18	1552		–12.70		9.10	0.02	0.12
27	99.6	0.73	0.00	–24.42	0.13	478	9	4.60	0.63	766	9	–14.64	0.27	18.86		0.12
25	92.8	1.41	0.23	–20.71	0.08	791	126	4.32	0.16	1220		–14.34		15.94		0.09
23	99.8	0.73	0.14	–21.07	0.05	465	63	3.71	0.20	768	93	–8.55	0.83	13.20		0.10
10	99.6	0.04	0.00	–20.26	0.09	76	2	2.85	0.03	82	0	–4.63	0.14	6.57	0.60	0.09

a SD = standard deviation of the parameter in the preceding column, in the same units. TOC (total organic carbon), TN (total nitrogen), and TRS (total reduced sulfur) are relative to the decarbonated residue. To convert to whole-rock quantities, these values would need to be multiplied by (1–carb.), where carb. = total carbonate content.

Bulk nitrogen isotope (δ^15^N_bulk_) values initially show an increase from +2.9‰ at the base to +5.8‰ at 35 cm, followed by a decrease to +3.9‰ at 84 cm (Figure 3A). The values then increase again to a plateau around +5.7‰ for the remainder of the section. Organic carbon isotopes (δ^13^C_org_) show a one-step decline from −20.7 ± 0.4‰ in the lower 25 cm to −25.2 ± 0.4‰ for the remainder of the section. One outlier with −29.5‰ occurs at 90 cm, i.e., in the same sample that also displays an anomalously higher C/N ratio. Isotopic ratios of total reduced sulfur (δ^34^S_TRS_) initially decrease from −4.6‰ at the base to −14.6‰ at 27 cm and then increase again to +2.7‰ at 100 cm. From then onward, the values scatter around 0‰. Carbonate-associated sulfate (δ^34^S_CAS_) shows a low isotopic value of +6.6‰ at the base, but for the remainder of the section, it is scattered around a mean of +17.1 ± 4.0‰.

### 3.2 Trace Element Trends

We focus on Yb_SN_/Pr_SN_ ratios, Y/Ho ratios (in units of g/g) as representative geochemical proxies for seawater conditions, and total Zr abundances as an almost immobile element representing the impact of detrital contamination on the geochemical budget of the stromatolitic carbonates in the leachates (Table 2). Yb_SN_/Pr_SN_ ratios increase from 1.1 at the base of the section to 3.3 at 44 cm (Figure 3B). This peak is only slightly above the local peak in δ^15^N_bulk_. From 44 to 84 cm, Yb_SN_/Pr_SN_ declines to a minimum of 1.6, before increasing again to values near or above 4 for the rest of the section (with one outlier at 140 cm). Y/Ho ratios display a similar pattern with an initial rise from a slightly superchondritic value of 30 at the base of the section to a strongly superchondritic value of 41 at 57 cm height, and then a decrease back to values around 30 between 64 and 84 cm. Zirconium abundances mostly cluster around a mean of 0.09 ± 0.03 μg/g, except for two values between 0.20 and 0.21 μg/g in the top two samples. It is possible that these two slightly Zr-enriched values reflect some detrital influence. With those two points, Zr is weakly correlated with Yb_SN_/Pr_SN_ (r^2^ = 0.21, *p* = 0.03, Figure 2G) and Y/Ho (r^2^ = 0.28, *p* = 0.01, Figure 2H), but this correlation disappears when those two points are excluded (Zr versus Yb_SN_/Pr_SN_: r^2^ = 0.00, *p* = 0.9, Figure 2G; Zr versus Y/Ho r^2^ = 0.18, *p* = 0.06, Figure 2H). Hence, the trends in Yb_SN_/Pr_SN_ and Y/Ho along the section are not controlled by detritus. Our data further highlight the importance of acetic acid leaching to avoid detrital contamination in subrecent carbonate samples in comparison to the 5N HNO_3_ leaching of Viehmann et al.,^10^ who observed partly significant detrital contamination of the geochemical budget of the carbonates.

**Table 2:** Zirconium and Rare Earth Element Plus Yttrium Concentrations in Carbonate Leachates Using 1M HAc^a^

Pos. [m]	Zr	La	Ce	Pr	Nd	Sm	Eu	Gd	Tb	Dy	Y	Ho	Er	Tm	Yb	Lu	Y/Ho	Yb_SN_/Pr_SN_
158	0.206	0.424	0.715	0.096	0.408	0.088	0.032	0.128	0.02	0.124	1.16	0.032	0.102	0.016	0.104	0.018	36.3	3.38
150	0.209	0.738	1.2	0.162	0.673	0.154	0.051	0.211	0.031	0.205	2.13	0.051	0.172	0.029	0.185	0.031	41.8	3.56
140	0.117	1.73	2.76	0.423	1.75	0.364	0.095	0.427	0.062	0.377	2.96	0.087	0.262	0.036	0.244	0.038	34.1	1.80
103	0.071	0.468	0.619	0.103	0.446	0.101	0.046	0.137	0.024	0.151	1.41	0.034	0.115	0.018	0.125	0.02	41.6	3.79
100	0.092	2.44	3.55	0.631	2.70	0.613	0.159	0.679	0.114	0.677	4.58	0.151	0.446	0.068	0.468	0.072	30.3	2.31
90	0.077	0.934	1.62	0.22	0.926	0.209	0.06	0.274	0.044	0.264	2.18	0.058	0.195	0.03	0.187	0.034	37.5	2.65
84	0.056	1.47	2.83	0.383	1.587	0.353	0.094	0.403	0.06	0.327	2.11	0.068	0.206	0.032	0.196	0.032	31.0	1.60
70	0.143	0.972	1.91	0.225	0.944	0.187	0.064	0.227	0.036	0.197	1.35	0.042	0.129	0.02	0.129	0.018	32.1	1.79
68	0.074	1.35	2.63	0.326	1.314	0.278	0.09	0.332	0.052	0.274	1.75	0.06	0.176	0.026	0.168	0.024	29.2	1.61
64	0.071	0.831	1.58	0.194	0.817	0.176	0.065	0.2	0.03	0.172	1.14	0.038	0.109	0.016	0.103	0.016	29.9	1.66
60	0.116	0.569	0.799	0.134	0.557	0.118	0.042	0.15	0.024	0.124	1.07	0.028	0.094	0.014	0.09	0.012	38.1	2.10
57	0.114	0.321	0.562	0.075	0.302	0.065	0.026	0.079	0.01	0.073	0.579	0.014	0.049	0.008	0.055	0.008	41.4	2.29
55	0.095	0.626	1.01	0.161	0.676	0.141	0.04	0.149	0.026	0.141	1.08	0.03	0.095	0.014	0.091	0.014	36.0	1.76
53	0.134	0.335	0.665	0.077	0.321	0.069	0.032	0.083	0.012	0.073	0.552	0.014	0.047	0.008	0.045	0.008	39.4	1.82
47	0.095	0.241	0.479	0.06	0.235	0.054	0.025	0.072	0.012	0.06	0.489	0.014	0.039	0.008	0.047	0.008	34.9	2.44
44	0.07	0.355	0.723	0.082	0.335	0.076	0.026	0.104	0.018	0.106	0.869	0.026	0.086	0.014	0.088	0.016	33.4	3.35
40	0.071	2.82	5.58	0.667	2.70	0.578	0.121	0.667	0.097	0.548	3.62	0.121	0.368	0.053	0.352	0.051	29.9	1.65
35	0.085	1.22	2.22	0.267	1.11	0.247	0.079	0.327	0.053	0.319	2.26	0.067	0.216	0.032	0.21	0.034	33.8	2.45
27	0.104	3.56	7.07	0.81	3.27	0.675	0.148	0.793	0.116	0.626	4.22	0.134	0.431	0.067	0.452	0.069	31.5	1.74
25	0.073	1.69	3.26	0.363	1.47	0.304	0.077	0.325	0.048	0.268	1.91	0.06	0.171	0.026	0.171	0.028	31.9	1.47
23	0.047	3.58	5.96	0.697	2.72	0.538	0.131	0.677	0.098	0.566	4.00	0.125	0.395	0.059	0.401	0.059	32.0	1.80
10	0.041	9.36	17.5	2.16	8.74	1.72	0.374	1.90	0.278	1.48	8.99	0.301	0.904	0.122	0.796	0.114	29.9	1.15

a All concentrations given in μg/g. SN = Shale normalized, where Post Archean Australian Shale (PAAS) is used for reference.

Yb_SN_/Pr_SN_ is poorly correlated with δ^34^S_CAS_ (r^2^ = 0.21, *p* = 0.03, Figure 2D) and TRS/TOC (r^2^ = 0.11, *p* = 0.44) but moderately correlated with δ^15^N_bulk_ (r^2^= 0.52, *p* = 0.0003, Figure 2C). The covariance between Yb_SN_/Pr_SN_ and δ^15^N_bulk_ is stronger if samples with Yb_SN_/Pr_SN_ ratios above 3.0 (which are statistical outliers relative to the remaining points) are taken out (r^2^ = 0.60), which probably indicates that N isotope ratios reach a maximum at this point and do not increase further as Yb_SN_/Pr_SN_ increases from 2.5 to nearly 4 (Figure 2C). However, more data are needed to verify this hypothesis. Y/Ho is also poorly correlated with δ^34^S_CAS_ (r^2^ = 0.02, *p* = 0.44, Figure 2F) and TRS/TOC (r^2^ = 0.1, *p* = 0.10) but moderately correlated with δ^15^N_bulk_ (r^2^ = 0.31, *p* = 0.01, Figure 2E).

## 4 Discussion

### 4.1 Trace Elements Support Fluctuating Ocean Connectivity

The low abundances of immobile Zr in our carbonate leachates (0.2 vs 200 μg/g in UCC, or 3 orders of magnitude lower) is evidence that the leaching protocol did not mobilize significant detrital silicate components. Furthermore, and as noted above, only the topmost samples lead to weak correlations between Zr and REY systematics, suggesting negligible detrital contamination on the overall geochemical compositions of the stromatolitic carbonates. The data can therefore be used to reconstruct REY patterns in the water column from which the carbonates precipitated, allowing us to draw inferences about the water chemistry. Our trace element record thus supports the results from Viehmann et al.,^10^ but the main difference is that our data extend all the way to the base of the section, providing a more complete record of ocean connectivity based on this proxy alone.

It is well established that open marine seawater is characterized by heavy REY enrichment relative to light REY in shale-normalized patterns.^30^ This relative enrichment is manifested in elevated Yb_SN_/Pr_SN_ ratios, along with other typical seawater-like features such as positive La_SN_, Gd_SN_, and Y_SN_ anomalies. Based on these proxies, Viehmann et al.^10^ identified an interval of restricted marine or possibly nonmarine conditions with low Yb_SN_/Pr_SN_ ratios and no significant to small anomalies between 40 and 100 cm stratigraphic height in the Rabenkopf section, bracketed by marine signatures with elevated Yb_SN_/Pr_SN_ ratios and strong positive La_SN_, Gd_SN_, and Y_SN_ anomalies. Our new REY data agree with this trend, but they also reveal low Yb_SN_/Pr_SN_ values at the base of the section (Figure 3B), meaning that conditions were also initially restricted marine to nonmarine, based on this proxy. A brief marine incursion likely caused the spike in Yb_SN_/Pr_SN_ and other typical seawater-like features at 44 cm, while restricted (possibly nonmarine) conditions reigned before (0–40 cm) and after (47–100 cm). As noted above, paleontological data have been interpreted as indicating continuously hypersaline conditions in a lagoon restricted from the open ocean,^21^ rather than waxing and waning seawater influence or even brackish conditions. However, the REY signatures seen here differ from those of hypersaline fluids and their precipitates.^10^ Paired with the lack of gypsum and other salt deposits in the stratigraphic section of the Ritzing area, the REY data thus suggest a lack of hypersaline conditions in the Rabenkopf section.

Also, Y/Ho ratios have previously been used as a marine/nonmarine indicator. While the effective 3+ ionic radii of Y and Ho are nearly comparable, making them geochemical twins, their differential capacity to create strong surface and solution complexes governs the fractionation of the pair in aquatic environments, leading to elevated Y/Ho ratios with increasing salinities.^30−32^ The ability of ancient carbonates to record the ambient seawater Y/Ho ratios makes the proxy a qualitative paleo-salinity proxy.^33^ In our data set, Y/Ho ratios broadly follow Yb_SN_/Pr_SN_ (Figure 3B), though with a slight offset, recording the long-term connectivity of the Oberpullendorf basin to the open ocean.

### 4.2 Sulfur Cycling Dominated by Seawater Input

Sulfur geochemistry has long been investigated and applied as a proxy for paleosalinity.^1,2^ The sulfate concentration of the modern ocean (28 mM) is roughly 170 times higher than that of average river waters (median 0.16 mM),^12^ which means that sediments with sufficient organic matter to stimulate sulfate reduction become relatively more enriched in reduced sulfur if they are influenced by seawater rather than freshwater. The resulting sulfide may bind to organic matter or precipitate as sulfide minerals, most commonly pyrite. Empirical calibrations suggest that freshwater sediments display TRS/TOC ratios below 0.1 by mass, while brackish and marine settings plot above 0.1 (ref (2)). In our sample set, TRS/TOC scatters around the threshold of 0.1, but the majority of the data points are slightly lower (Figure 2B). We find no strong enrichments in TRS that would be indicative of sulfidic (euxinic) marine conditions.^34^ Instead, the relatively low TRS/TOC ratios and the strong correlation with TOC suggest that sulfate levels in the water column were probably lower than those in the open ocean and that most sulfide is organic-bound. However, the absence of stratigraphic trends (Figure 3A) means that this proxy is not as sensitive to the transition from open-marine to restricted conditions as the trace elements (this study, ref (10)). In other words, this proxy is similarly invariable as the paleontological data across the section,^21^ though not showing evidence of hypersalinity in the form of TRS/TOC ratios well above the marine/nonmarine threshold.

The isotopic composition of carbonate-associated sulfate should theoretically be a good indicator of marine versus nonmarine settings because seawater is enriched in ^34^S/^32^S (+21‰^11^) compared to river waters (+4.4‰^12^). In the Miocene, seawater sulfate values have been constrained to +22 ± 0.5‰.^29^ The composition of average Miocene rivers is unknown but on average likely similar to the modern value given the generally similar crustal configuration and age distribution. Of course, the riverine δ^34^S composition of sulfate may also vary spatially,^12,35^ but if we assume that this basin received riverine sulfate with a composition close to the global average, then a strong freshwater influence may be evident in the form of a lower δ^34^S_CAS_ value. We do indeed see a low δ^34^S_CAS_ value at the base of the section (Figure 3A), which may indicate a fluvial influx of sulfate. If so, the subsequent increase in δ^34^S_CAS_ may reflect an increasing marine input and relatively higher salinity (though never reaching the fully marine endmember). Importantly, this interpretation is consistent with the trace element record for this lower part of the section, which also indicates a *relative* increase in seawater input.

However, for the remainder of the section, δ^34^S_CAS_ does not replicate the trends seen in Yb_SN_/Pr_SN_ and other typical seawater proxies (this study, ref (10)), meaning that like TRS/TOC this proxy fails to capture the environmental transitions that affected the trace element record. The lack of correlation between Yb_SN_/Pr_SN_ and δ^34^S_CAS_ (Figure 2D) further supports this conclusion. We also note that the mean value for the remainder of the section (+17.1 ± 4.0‰.) remains below that inferred for Miocene seawater. It is possible that the CAS data were affected by postdepositional sulfide oxidation to sulfate. If such diagenetic sulfate was incorporated into secondary carbonate phases, it may have lowered the preserved δ^34^S_CAS_ value of bulk rocks. We cannot rule out this possibility, but given the quite systematic isotopic depletion, this possibility is perhaps less likely. Instead, the systematically lower δ^34^S_CAS_ value compared to contemporaneous seawater perhaps indicates mixing between freshwater and seawater sulfate throughout the section. If so, then like TRS/TOC, δ^34^S_CAS_ is inconsistent with hypersalinity inferred from paleontological observations,^21^ for which we would expect δ^34^S_CAS_ values closer to the Miocene seawater value of +22 ± 0.5‰.^29^

Support for the presence of a moderate sulfate reservoir comes from the δ^34^S_TRS_ data. Reduced sulfur is typically depleted in ^34^S/^32^S relative to sulfate because microbial sulfate reduction imparts a significant isotopic fractionation of up to 70‰,^36^ where the sulfide becomes lighter and residual sulfate becomes isotopically heavier. When the sulfate reservoir is locally depleted under closed-system conditions, Rayleigh distillation drives residual sulfate and thus newly formed sulfide to higher ^34^S/^32^S ratios that may approach or even exceed the composition of the original sulfate. This phenomenon is often observed in diagenetic pore waters.^37^ An additional complication in our sample set is that most, if not all, reduced sulfur may be organic-bound rather than pyrite-bound, as suggested by the strong correlation between TOC and TRS. Due to the low TRS abundance and the small amount of residue remaining after decarbonation, we were not able to extract pyrite separately. Organic matter can acquire sulfur in two ways: through binding secondary sulfide during diagenesis (sulfurization) or through active assimilation of sulfate from the water column. These two pools may be isotopically distinct, making it challenging to interpret δ^34^S_TRS_ data dominated by organic components. We can therefore not discuss this data set in great detail; however, we point out that δ^34^S_TRS_ is offset from δ^34^S_CAS_ by 24.4 ± 6.5‰ on average (Figure 3A), which is lower than the maximum possible offset of 70‰,^36^ but consistent with persistent availability of at least moderate sulfate levels in this local environment (>0.2 mM^38^), such that net reduction did not go to completion. It is also possible that a large proportion of reduced sulfur was rapidly reoxidized within the same environmental setting without significantly affecting the δ^34^S record. Such a “cryptic sulfur cycle” has been described from TRS-poor gypsum deposits of Messinian age whose δ^34^S tracks the composition of contemporaneous seawater.^39^ Local variability in the degree of sulfide formation and reoxidation may contribute to the variability observed in δ^34^S_CAS and_ δ^34^S_TRS_. In any case, our results differ from modern sulfate-poor lakes (0.1–0.35 mM), where sulfide in sediments trends toward the isotopic composition of the available sulfate.^40^ Hence despite the uncertainties created by potential organic contributions to the TRS pool and by potential Rayleigh effects on δ^34^S_CAS_, the δ^34^S_TRS_ data are overall consistent with at least some marine influence throughout the section, as inferred from the δ^34^S_CAS_ and TRS/TOC ratios.

### 4.3 Carbon Isotope Sensitivity to Ecology

Organic carbon isotopes are fractionated during CO_2_ fixation, where organic matter becomes lighter relative to residual dissolved CO_2_. Variations in the δ^13^C_org_ value may thus reflect either changes in the isotopic composition of aqueous CO_2_ or differing enzymatic pathways in the fixation process. The large atmospheric CO_2_ reservoir connects all water bodies; therefore, the isotopic composition of dissolved CO_2_ may not necessarily differ between seawater and river waters. In practice, however, oxidation of terrestrial organic carbon to dissolved inorganic carbon (which includes CO_2_), paired with sluggish atmospheric equilibration, may lead to lower ^13^C/^12^C ratios in river water,^41^ which could theoretically translate into lower δ^13^C_org_ values if CO_2_-fixation pathways are the same.

In our data set, however, δ^13^C_org_ shows no response to the inferred marine incursion at 44 cm, nor to the subsequent return to restricted conditions at 44–100 cm. A more plausible explanation for the observed trend in δ^13^C_org_ (Figure 3B) is perhaps a shift in the ecosystem at 27 cm toward organisms that impart larger fractionations in ^13^C/^12^C. This interpretation is broadly consistent with the observed switch from macro-fossiliferous carbonates to stromatolites.^21^ Such a shift may have been induced by a change in water composition toward more marine-dominated conditions, as suggested by the δ^34^S_CAS_ data. The single outlier in δ^13^C_org_ at 90 cm likely represents terrestrial biomass, such as plant debris, that was washed into the basin. This interpretation is supported by the high C/N ratio of this sample, which is more typical for plant tissue.^42^ Organic carbon isotopes and C/N ratios do therefore track the evolving ecology of the setting and a shift toward different CO_2_-fixation pathways in microbial mats compared to macrofauna, but they do not show any discernible response to the restricted interval at 44–100 cm indicated by depressed Yb_SN_/Pr_SN_ ratios and are, thus, not a sensitive proxy for seawater versus freshwater dominance.

### 4.4 Nitrogen Isotope Response to Intervals of Basin Restriction

As described above, carbonate-associated sulfur isotopes (δ^34^S_CAS_) suggest a shift toward more marine-influenced conditions from approximately 25 cm upward, and the organic carbon isotope data may reflect a small ecological change associated with this transition, consistent with the replacement of macrofossils by stromatolites. However, neither of the two proxies captures the weakened seawater influx at the base of the section and in the 47–100 cm interval inferred from trace elements, specifically, Yb_SN_/Pr_SN_ ratios (Figure 3). In contrast, the nitrogen isotope data do show a response and a good covariance with Yb_SN_/Pr_SN_ (Figure 2C).

The initial increase from 2.9‰ to 5.8‰ in δ^15^N_bulk_ coincides with the increase in δ^34^S_CAS_ and thus perhaps suggests increasing input of marine nitrate with a composition of around 6‰, similar to the modern ocean.^18^ We note again that marine nitrate may be variable in δ^15^N, but a value of 6‰ is a reasonable approximation, given that it is a strong mode in the modern ocean.^17^ This overall interpretation of the δ^15^N_bulk_ data is consistent with our new Yb_SN_/Pr_SN_ data. The isotopic composition of nitrate can be archived in organic matter-bearing sediments via the burial of biomass that assimilated this nitrate from the water column.^17^ If so, then the drop in δ^15^N_bulk_ from 5.8‰ at 35 cm to 3.9‰ at 84 cm (Figure 3A) may be a return to conditions where marine nitrate was less available such that N_2_-fixing organisms (whose composition falls near 0‰) became relatively more prevalent, similar to the base of the section. This interpretation would match the trace element data showing continuously decreasing seawater-like REY systematics within this interval (Figure 3B). As noted earlier, the isotopic composition of preindustrial riverine δ^15^N_NO3_ is unknown, but Holocene lake sediments fall around a mean of +2.2‰.^20^ This relatively low mean value, i.e., lower than marine nitrate, reflects a significant proportion of biological N_2_ fixation due to nitrate limitation in freshwater lakes. A similar explanation may apply in our data set, where the decrease in δ^15^N_bulk_ values likely reflects a decrease in nitrate availability and therefore enhanced N_2_ fixation—either in situ within the microbial mats that constitute the stromatolites or in fluvio-lacustrine waters upstream of the basin, which then generated a flux of isotopically light nitrate via river waters into the study site. A combination of these two processes may explain the small-scale variability in the data. But to first order, this interpretation would imply that marine nitrate levels were restored above 80 cm, where δ^15^N_bulk_ returns to values near +6‰, also in agreement with the trace element record showing a return from nonseawater-like REY_SN_ patterns to typical seawater-like REY_SN_ systematics (Figure 3).

We stress that this overall interpretation of the nitrogen isotope record does not change if diagenetic alteration is considered. It has been shown that δ^15^N_bulk_ values in sediments tend to increase by a few permil in oxic settings.^43^ The sediments from this study site were likely deposited in an oxic environment, as indicated by Ce anomalies within the carbonate.^10^ If there was a significant diagenetic increase in δ^15^N, this process would have affected all samples and would not have created the observed stratigraphic patterns.

### 4.5 Residence Time Considerations for Seawater Proxies

At first glance, our isotopic data appear to be conflicting with each other and with previous paleontological interpretations. REY and δ^15^N_bulk_ suggest fluctuating strength in marine input along the section, while sulfur isotopes and abundances suggest a nearly continuous marine connection, though possibly with lower sulfate levels than those in the open ocean. In contrast, the presence of microbialites and *Ammonia beccerii* occurrences has been interpreted as reflecting hypersalinity, although, as noted in Section 2, a brackish water interpretation is also viable for these particular organisms.

The most parsimonious explanation for these conflicting results is the differences in the residence time of the various chemical species. Residence time is defined as the ratio of a reservoir size over the exchange flux, and it determines how fast an element is removed from an environment. In the modern global ocean, sulfate has a residence time of approximately 8.7 × 10^6^ years, which is over 3 orders of magnitude longer than the residence time of nitrate with 3.7 × 10^3^ years.^14^ It is also much longer than the residence times of Yb (2.2 × 10^3^ years) and Pr (5 × 10^2^ years) (and other REEs), which are similar to or slightly shorter than the general ocean mixing time of 1500 years. Hence, a seawater signature in the form of sulfur takes longer to remove from the environment and may therefore linger during brief periods of heightened freshwater influx. We cannot know the residence times of elements within the Oberpullendorf Basin; however, the global ocean, for which residence times are well constrained, can serve as an example for illustrating the effects that the residence time has on geochemical proxies.

**Figure 4:**
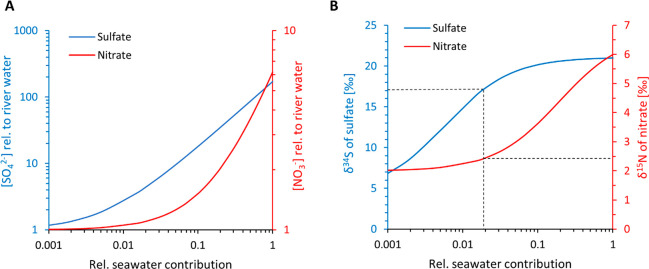
Mixing calculations for seawater with 28 mM sulfate, δ^34^S_SO42–_ = +21‰, and 31 μM nitrate, δ^15^N_NO3–_ = +6‰, and river water with 0.16 mM sulfate, δ^34^S_SO42–_ = +4.4‰, and 5 μM nitrate, δ^15^N_NO3–_ = +2.2‰. See the text for references. (A) Concentrations in dissolved sulfate (blue) and nitrate (red) as a function of seawater input. (B) Corresponding isotopic composition of sulfate (blue) and nitrate (red) in the mixture. We stress that these calculations do not account for potential biological processes that may perturb the isotopic composition of sulfate and nitrate during mixing. Such local effects may increase isotopic variability.

These effects can be illustrated quantitatively if we assume seawater concentrations of 28 mM for sulfate and 31 μM for nitrate and river water concentrations of 0.16 mM and 5 μM, respectively. With these numbers, for example, the sulfate concentration of a brackish mixture with only 10% seawater would already be 18 times higher than that of the freshwater endmember. In contrast, the nitrate concentration would be only 1.5 times higher (Figure 4A). A δ^34^S value for sulfate of 17‰, as observed for our CAS data above the base of the section (Figure 3), could be achieved with less than 2% seawater input (assuming that these values do not reflect diagenetic sulfide oxidation). Such a low salinity occurs, for example, in estuaries around the modern Baltic Sea.^44^ In comparison, the δ^15^N value of nitrate of such a weakly saline mixture would still fall close to the freshwater endmember (Figure 4B, dashed black line). The sulfate concentration of a fluid with 2% seawater would be 3.5 times higher than in river water, or approximately 0.7 mM, which is sufficient for microbes to express an isotopic fractionation during sulfate reduction.^38^ It would contain 5.5 μM nitrate and thus merely 10% more than freshwater. Within microbial mats, where nitrate is rapidly consumed, this may stimulate N_2_ fixation.

Our results may help reconcile discrepancies between geochemical and paleontological data in this section. The fossil assemblage at the top of the second sedimentary depositional cycle of the middle Badenian in Oberpullendorf Basin, generally speaking, reveals a digression from normal marine conditions above 20 cm with the onset of microbialites, but the reasons for this digression and its specific nature cannot be inferred from fossils alone. Previous interpretations favored hypersaline conditions in a lagoonal environment restricted from the open ocean,^21^ where high salinity would imply high concentrations of sulfate and nitrate, as these are part of the salt inventory of seawater. However, δ^15^N, REY, and other trace elements suggest a temporary cessation of open marine exchange between 47 and 100 cm and reflooding above 100 cm, while the sulfur data reveal moderate marine influence throughout, though possibly with lower sulfate levels than those in the open ocean. Hence, the geochemical data do not support the paleontological inference of a hypersaline lagoonal environment, but they support the notion of restriction from the open ocean. Restriction was strongest at 47–100 cm. Above and below this interval, relatively more seawater was able to penetrate, but conditions may have been somewhat brackish. The inferred fossil assemblage may thus reflect restricted, brackish-water conditions rather than hypersalinity, for which we did not find any geochemical evidence. The inferred salinity fluctuations along the section may have exerted stress on local fauna and thus contributed to the exclusion of macroorganisms and the proliferation of foraminifera that are capable of handling rapid environmental change.

**Figure 5:**
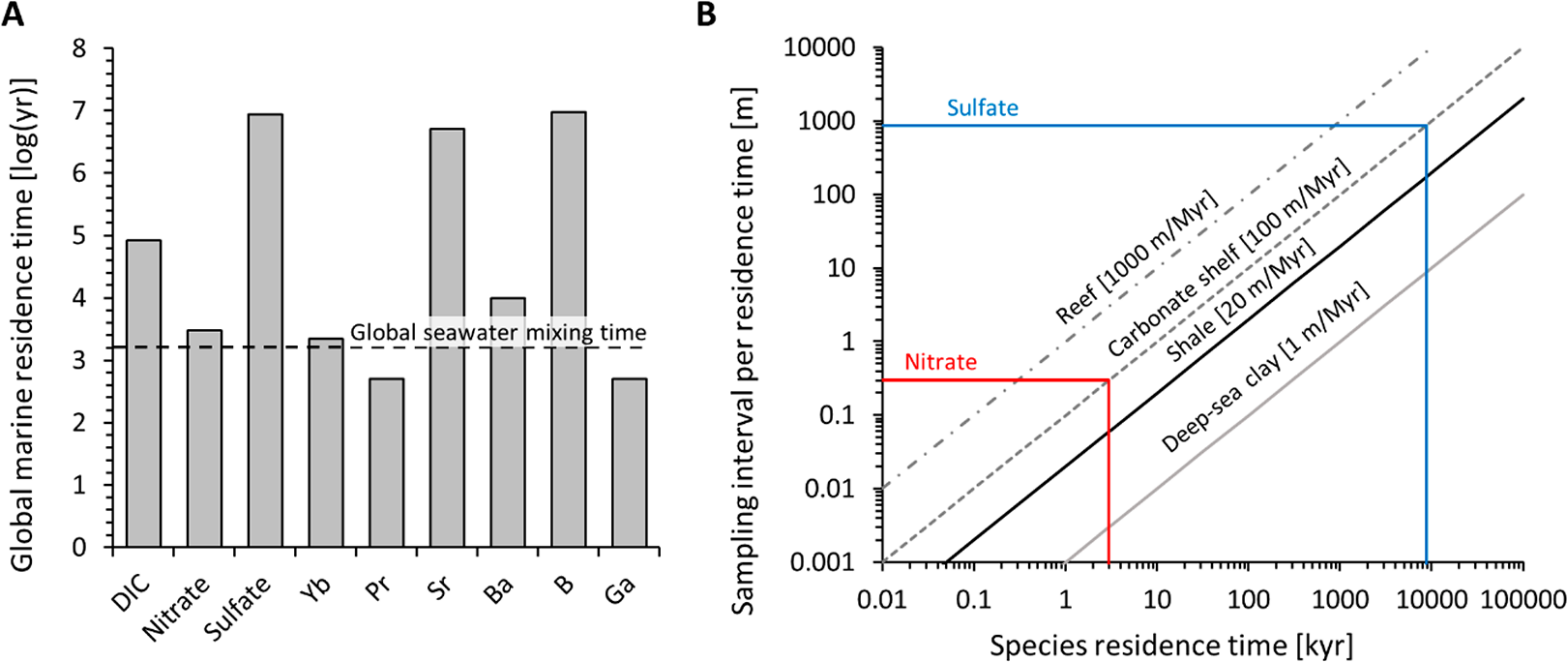
(A) Residence times of several relevant species that are used as seawater proxies, taken from Henderson and Henderson.^14^ (B) Calculation of required sampling density per residence time as a function of sediment deposition rate, after Johnston and Fischer.^45^

Our analysis also carries an important lesson for future studies that rely on traditional stable isotopes and elemental ratios to determine if ancient sediments were laid down in marine or nonmarine conditions. Where sulfate is used as a sole proxy, brief nonmarine intervals during which a basin was disconnected from the ocean may be missed. A similar problem could arise when Sr/Ba or B/Ga ratios are used as proxies.^2^ Both Sr and B have long residence times in seawater, comparable to those of sulfate (Figure 5A), and may thus linger in the environment long after connectivity to the open ocean has been interrupted. Conversely, these proxies are very sensitive for detecting marine influx into otherwise nonmarine settings. In contrast, nitrate and REE all have short residence times of a few thousand years or less (Figure 5A), meaning that they and their isotopes are more likely to capture short-lived events such as periodic marine or nonmarine stages. Dissolved inorganic carbon (DIC) has an intermediate residence time of 83,000 years, which in the case of our study site may have been long enough to buffer against freshwater influx.

Lastly, the differing residence times of these proxies also need to be considered in the sampling density. In our study site, the restricted interval comprises the basal few centimenters of the section and then the interval from 47 to 100 cm in stratigraphic height, where Yb_SN_/Pr_SN_ ratios and δ^15^N_bulk_ values are low. Fluctuations between restricted and open-marine conditions thus occur on decimeter scale. As first illustrated by Johnston and Fischer,^45^ the required sampling density for obtaining one sample per residence time is a function of the sediment deposition rate. For example, in a carbonate shelf, where the sediment deposition rate is approximately 0.1 mm·yr^–1^ (100 m·Myr^–1^), a species with a residence time of a few thousand years, such as nitrate or REE, would require one sample every few decimeters to capture each residence time once (Figure 5b). Similar growth rates of 0.01–0.5 mm·yr^–1^ (10–500 m·Myr^–1^) have been inferred for stromatolites at modern Shark Bay.^46,47^ This agrees roughly with our sampling density, which appears to have been adequate for capturing the nonmarine interval in both nitrate and Yb_SN_/Pr_SN_ ratios (Figure 3). As noted above, we do not know the exact deposition rate at our study site due to depositional hiatuses over the proposed 200–600 kyr time span.^21^ If the deposition rate was closer to that of a carbonate reef (1 mm·Myr^–1^ or 1000 m·Myr^–1^), we would not necessarily expect changes in δ^15^N_bulk_ over a few decimeters because it would take over 1 m of deposition before the nitrate reservoir of the basin has turned over. This suggests that the true deposition rate was closer to that of a carbonate shelf. In either case (reefs or carbonate shelf depositional rates), significant changes in sulfur isotopes would only be expected on the km scale due to the long residence time of sulfate in seawater, which further illustrates the limitation of the sulfur proxy for tracking short-lived nonmarine intervals.

Importantly, the quantitative aspects presented herein likely differ in each basin, where the residence time of chemical elements may differ from that of the global ocean. They likely also differed in earlier times in Earth’s history when seawater was largely anoxic and thus depleted in species such as sulfate and nitrate.^48^ For example, sulfate concentrations in the Archean ocean may have been over 3 orders of magnitude lower than today,^49^ and nitrate was probably limited to rare oxygen oases in surface waters.^50,51^ At that time, sulfate and nitrate would likely have acted as nonconservative species in seawater with markedly shorter residence times and response times to nonmarine conditions. Both nitrate and sulfate concentrations increased in seawater around the time of the Paleoproterozoic Great Oxidation Event^52,53^ and probably reached modern levels in the Neoproterozoic or Phanerozoic during the second rise of oxygen.^54,55^ In a previous study, we showed that late Proterozoic marine sulfate levels (at ∼1.1 Ga) were high enough to leave a significant mark in brackish environments, where trace elements and nitrogen isotopes revealed a nonmarine influence.^56^ Hence the modern hierarchy of proxy response time was perhaps established by that point; however, care is nevertheless required in deep-time studies at variable stratigraphic resolution.

## 5 Conclusions

Our results demonstrate the effects of residence time on the response of proxies to environmental conditions. Sulfur-based proxies (both isotopes and TOC/TRS ratios) are insensitive to the short-lived episode of restricted marine influence during the middle Badenian in the Oberpullendorf Basin, whereas REY (e.g., Yb_SN_/Pr_SN_ ratios) and nitrogen isotopes respond synchronously, consistent with their similar residence times. Our model calculations show that only a few percent of seawater are sufficient for a basin to appear “marine” in terms of sulfur chemistry, while such a mixture would still look “nonmarine” in nitrogen isotope space. The same principle would likely apply to other seawater proxies that are in use such as B/Ga ratios or Sr/Ba ratios. With regard to the Oberpullendorf Basin, our results overall do not support the paleontological inference of a hypersaline lagoonal setting throughout the studied section at any point; however, they do support the idea of restriction from the open ocean, which may have been a driver of macrofaunal exclusion at this site. Lastly, we conclude that the choice of proxies must consider the effects of residence time, and sampling densities must be adjusted to optimize analytical campaigns. Short-lived proxies such as REY or nitrogen isotopes may be more sensitive to short-lived marine incursions or freshwater intervals, but they require a high sampling density such that each residence time can be sampled at least once. Extra care must be taken in deep time, where the composition of seawater and hence the residence time of dissolved species may have differed markedly from modern day.

## Supporting Information Available

Detailed description of sample preparation and analytical protocols (PDF)
