# Supplementary material for: Exploring the Effects of Residence Time on the Utility of Stable Isotopes and S/C Ratios as Proxies for Ocean Connectivity
Source: ACS Earth Space Chem. 2023 Jul 8;7(7):1337–49. doi: 10.1021/acsearthspacechem.3c00018 (PMC10364137; doi:10.1021/acsearthspacechem.3c00018)
Supplement: Supplementary file 1 — sp3c00018_si_001.pdf [file sp3c00018_si_001.pdf]

Supplementary Information for:

Exploring the effects of residence time on the utility of stable isotopes and S/C ratios as proxies for ocean connectivity

Eva E. Stüeken[1,*], Sebastian Viehmann[2,3], Simon V. Hohl[4]

1. University of St Andrews, School of Earth & Environmental Sciences, Bute Building, Queen's Terrace, St Andrews, Fife, KY16 9TS, United Kingdom
2. Department of Lithospheric Research, University of Vienna, Josef Holaubek-Platz 2, 1090 Vienna, Austria
3. Institute of Mineralogy, Leibniz University Hannover, Callinstraβe 3, 30167 Hannover, Germany
4. State Key Laboratory of Marine Geology, Tongji University, Siping Road 1239, Shanghai, 200092, P.R. China
* corresponding author: ees4@st-andrews.ac.uk

S1. Sample Preparation for CNS analyses

We obtained off-cuts from the same stromatolite hand specimens and sampling regions as described in Viehmann *et al.*[1]. Each stromatolite sample comprised multiple laminae, which was necessary to have enough material for analyses. The sampling density along the 1.5 m thick stratigraphic section was on the order of a few cm to dm with the aim of capturing as much information as possible about species with short residence times in the water column. Approximately 10 g of rock powder were weighed into glass beakers, which had previously been acid-washed (reagent grade 2 N HCl overnight) and baked (500 °C overnight). The powder was mixed with 50 ml of 10 wt. % NaCl solution (reagent grade) to remove any soluble sulfate that may have precipitated during sediment deposition or formed from pyrite oxidation during atmospheric exposure. The samples were swirled a few times during the day and then left to settle overnight. On the following day, the NaCl brine was decanted, being careful not to lose any sediment, and the residual sediment was mixed with 100 ml DI-water (18.2 MΩ·cm^{-1}) to wash out remaining salts. The beakers were again swirled throughout the day and left overnight for the sediment to settle. The water was carefully decanted the following day. Then 2 M HCl was added in small quantities and swirled slowly. A total of 150 ml of acid was added until all effervescence had stopped, indicating the complete dissolution of carbonate. The acid, which now contained carbonate-associated sulfate (CAS) in solution, was then quickly transferred into 50 ml Falcon centrifuge tubes with the suspended insoluble residue and centrifuged at 3900 rpm for 5 min. To be able to centrifuge the entire sample at once, it was required to spread each sample out over three centrifuge tubes. The liquid supernatant (HCl with dissolved CAS) was decanted into a fresh beaker, where supernatants from multiple tubes of the same sample were re-combined. This solution was mixed with 20 ml of 0.5 M $BaCl_2$ and left overnight to allow $BaSO_4$ to form and precipitate. To collect the $BaSO_4$, the solution and

precipitate were transferred into fresh 50 ml Falcon centrifuge tubes and centrifuged at 3900 rpm for 10 min. The solution was decanted into a waste container, and the solid $BaSO_4$ residue was dried in an oven at 70 °C for subsequent isotopic analyses. For comparison, see, for example ref. 2.

The solid sediment residues after HCl-treatment of the rock powder (see above) were mixed with 20 ml of 1 N HCl, resuspended, and left overnight with loose caps, such that any residual carbonate was able to dissolve. The following day, the samples were centrifuged (3900 rpm for 10 min), the acid was decanted, and the sediment residue was washed three times with DI-water. The washed sediment was then placed into a freezer, and once frozen, transferred into a freeze-drier for five days to remove all moisture. The mass difference between the dried decarbonated residue and the initial rock powder was used to quantify the total carbonate content.

S2. CNS Isotopic Analyses

The decarbonated rock powder was analysed for total organic carbon (TOC), total nitrogen (TN), and total reduced sulfur (TRS). Approximately 10-40 mg of powder were weighed into tin capsules (8 mm x 5 mm in cross-section). For TRS analyses, 7-10 mg of V_2O_5 were added to the rock powder to serve as combustion aid. For the barite extracts, approximately 0.2 mg of $BaSO_4$ was used and mixed with 0.5 mg of V_2O_5. The filled capsules were analysed with an EA Isolink coupled via a Conflo IV to a MAT253 IRMS (all from Thermo Fisher Scientific). For carbon and nitrogen analyses, the EA was equipped with two reactor columns, one containing Cr_2O_3 and cobaltous/cobaltic silvered oxide (held at 1020 °C) and one containing pure Cu wire (held at 650 °C). A single reactor packed with WO_3 and electrolytic Cu (held at 1020 °C) was used for sulfur analyses. In both cases, water vapor was trapped with magnesium perchlorate at room temperature. The GC oven of the EA was ramped up to 240 °C during each analysis to ensure complete elution of all gases.

All data are expressed in standard delta notation ($\delta = [R_{sample}/R_{standard} - 1] \times 1000$), where R = $^{13}C/^{12}C$ for $\delta^{13}C$, $^{15}N/^{14}N$ for $\delta^{15}N$, and $^{34}S/^{32}S$ for $\delta^{34}S$, respectively. The standards are VPDB for carbon, atmospheric air for nitrogen, and VCDT for sulfur. Carbon and nitrogen isotopes were calibrated with the international reference materials USGS-40 and USGS-41. USGS-62 was used as a quality control standard, and the measured data ($\delta^{13}C$ = -14.8 ± 0.1 ‰, $\delta^{15}N$ = +20.2 ± 0.1 ‰, n = 9) were in good agreement with expected values ($\delta^{13}C$ = -14.79 ± 0.04 ‰, $\delta^{15}N$ = +20.17 ± 0.06 ‰). Sulfur isotopes were calibrated with IAEA-S2 and IAEA-S3. NBS-127 was used for quality control, and it also agreed well ($\delta^{34}S$ = +21.4 ± 0.3 ‰, n = 10) with the expected value (+21.17). Blanks resulting from empty tin capsules were subtracted from each sample and standard by mass balance. A subset of samples was analysed in duplicates, and the average reproducibility was 0.1 ‰ for $\delta^{13}C_{org}$ and 0.3 ‰ for $\delta^{15}N_{bulk}$, $\delta^{34}S_{TRS,}$ and $\delta^{34}S_{CAS}$. TOC, TN, and TRS abundances were quantified using the peak areas of the IRMS data, calibrated with a series of standards with varying masses. The average relative errors of the abundances for our samples were 5 % for TOC, 4 % for TN, and 3 % for TRS. The CAS abundance could not be accurately quantified because the barite residues in the centrifuge tubes were too small to obtain accurate weights of the total mass. We were not able to obtain a $\delta^{15}N_{bulk}$ value for two samples due to the comparatively low N-concentration and a small amount of sample powder remaining after decarbonation. For one sample, insufficient CAS was obtained for analysis.

S3. Trace element leaching and analyses

To ensure that the trace element trends reported by Viehmann et al. [1] are reproducible in the same sample aliquots that were used for the isotopic analyses in this study, we repeated the trace element work with an acetic-acid leaching protocol. Circa 50 mg sample powder aliquots were weighed into pre-cleaned 15 ml centrifuge vials washed with 10 ml DI water, followed by 1M NaOH and centrifugation at 4500 rpm for 5 mins. The washed powders were then treated with 1 M ultra-pure glacial acetic acid (HAc), ultrasonicated for 15 mins, and reacted overnight before they were ultrasonicated again and centrifuged at 4500 rpm for 5 mins. The leached solutions were then separated from the residues and pipetted into the second set of centrifuge vials for storage. This solution was used as a carbonate leachate stock solution. An aliquot of the stock solution was diluted into 10 ml of 2 % HNO_3 for trace element analysis using a dilution factor of ~1:2000. Trace element analyses were performed on an Agilent 7900 quadrupole ICP-MS equipped with a collision/reaction cell and using online Rh doping for drift correction. Analytical precision on trace element concentrations was <5 %, expressed as the relative standard deviation (1 SD) of repeated analyses of a bulk digestion solution of the GSD-9 geologic reference material. Analyses of carbonate reference material JDo-1 determined the accuracy of our method. Oxide-formation rates were below 3 %, but additional offline BaO interference corrections were applied following Dulski[3] due to high Ba concentrations in the leached fraction. REY patterns obtained in carbonate leachates have been normalized to Post Archean Australian Shale (PAAS[4]).

S4. Safety statement

No unexpected or unusually high safety hazards were encountered.

Supplementary References

1. Viehmann, S.; Kujawa, R.; Hohl, S. V.; Tepe, N.; Rodler, A. S.; Hofmann, T.; Draganits, E., Stromatolitic carbonates from the Middle Miocene of the western Pannonian Basin reflect trace metal availability in microbial habitats during the Badenian Salinity Crisis. *Chemical Geology* **2023,** *618*, doi: 10.1016/j.chemgeo.2023.121301.
2. Theiling, B. P.; Coleman, M., Refining the extraction methodology of carbonate associated sulfate: Evidence from synthetic and natural carbonate samples. *Chemical Geology* **2015,** *411*, 36-48.
3. Dulski, P., Interferences of oxide, hydroxide and chloride analyte species in the determination of rare earth elements in geological samples by inductively coupled plasma-mass spectrometry. *Fresenius' Journal of Analytical Chemistry* **1994,** *350* (4), 194-203.
4. Taylor, S. R.; McLennan, S. M., *The continental crust: its composition and evolution*. Blackwell: 1985.
